# High Intensity Interval Training Leads to Similar Inflammatory Activation as Seen With Traditional Training in Chronic Heart Failure

**DOI:** 10.3389/fcvm.2021.752531

**Published:** 2022-02-08

**Authors:** Arlana G. Taylor, Andrew I. Ignaszewski, Shannon S. D. Bredin, John S. Hill, Erin M. Shellington, Darren E. R. Warburton

**Affiliations:** ^1^Cardiovascular Physiology and Rehabilitation Laboratory, University of British Columbia, Vancouver, BC, Canada; ^2^Healthy Heart Program, St. Paul's Hospital, Vancouver, BC, Canada; ^3^Indigenous Health and Physical Activity Program, University of British Columbia, Vancouver, BC, Canada; ^4^Laboratory for Knowledge Mobilization, University of British Columbia, Vancouver, BC, Canada; ^5^University of British Columbia James Hogg Research Centre, Institute of Heart and Lung Health, Vancouver, BC, Canada; ^6^Experimental Medicine Program, University of British Columbia, Vancouver, BC, Canada

**Keywords:** heart failure, exercise, training, inflammatory markers, cytokines, heart failure, interval training, cardiac rehabilitation

## Abstract

**Background:**

Inflammatory activation has been associated with the severity and progression of chronic heart failure (CHF). Although cardiac rehabilitation is an important therapy, acute bouts of exercise may lead to increases in pro-inflammatory cytokines with exercise intensity mediating these changes.

**Objective:**

To evaluate the acute inflammatory response in patients living with CHF during a randomized trial following Steady State (SS) or High Intensity Interval (HIIT) training.

**Methods:**

Patients living with CHF (*n* = 14) were stratified (for body mass and aerobic power) and randomized into SS and HIIT cycle exercise. The HIIT exercise training involved 2 min work:recovery phases at 90:40% heart rate reserve. The SS exercise training involved continuous exercise at 65% of heart rate reserve (matched total work). Acute inflammatory markers were evaluated (via ELISA) at baseline, immediately following the bout, and at 6, 24, and 48 h post-exercise.

**Results:**

There was limited differences in the changes in inflammatory biomarkers across time between the HIIT and SS groups. Both groups experienced a significant (*p* < 0.05) change in Interleukin-6 immediately post-exercise.

**Conclusions:**

A single bout of HIIT or SS does not result in excessive inflammatory activation in CHF patients. Acute HIIT and SS result in similar changes in inflammatory markers. These findings have important implications for exercise training and rehabilitation programs in persons living with CHF.

## Introduction

Inflammatory activation with increased plasma/serum cytokine levels has been described as an important factor for the progression of chronic heart failure (CHF) (1, 2). Cytokines appear to act as catabolic factors in the pathogenesis of skeletal muscle wasting and cardiac cachexia (3–5). This has important implications as muscle mass is an important determinant of exercise and functional capacity (6). Moreover, the progressive loss of muscle mass, cachexia (i.e., weight loss due to an underlying illness), and low levels of muscular strength are strong predictors of the risk for premature mortality (5, 7, 8). Emerging research has demonstrated the important relationship between cytokines, health, and cardiovascular fitness. Lower levels of physical activity, functional status, and/or cardiovascular fitness have been associated with higher levels of inflammation in apparently healthy individuals (9) and persons living with chronic medical conditions (10).

Exercise training remains an important therapeutic intervention in the management of CHF improving exercise capacity, Quality of Life, and various neurohormonal abnormalities (11–14). Acute bouts of exercise can lead to increases in pro-inflammatory cytokines (15–17). In contrast, there is evidence that longer duration (6 weeks to 4 months) exercise training trials may lead to small reductions in pro-inflammatory markers (18–20). However, the evidence in this field remains unclear likely owing to a variety of factors, such as high heterogeneity of participant populations and/or exercise programs studied, low exercise training adherence, and different clinical characteristics or outcomes (14). For instance, some studies have demonstrated that strenuous high intensity physical activity can cause sub-clinical skeletal muscle injury with the potential for an excessive inflammatory reaction and immune suppression (in healthy individuals) (15). A recent 12-week exercise training study revealed that pro-inflammatory markers may be increased in healthy adults following exercise (21). Whereas, another investigation (18) revealed that 12 weeks of group-based cardiac rehabilitation (involving both high intensity interval (HIIT) and moderate intensity steady state (SS) training groups) resulted in a reduced inflammatory state in persons living with CHF.

In the presence of an increased baseline of inflammatory factors, it is possible that even small amounts of physical activity can acutely increase plasma/serum cytokines in extremely deconditioned individuals (such as those living with CHF). Further to this, persons living with CHF who have relatively low inflammatory markers may improve their cardiorespiratory fitness significantly more than those with high inflammatory markers following an exercise program that includes interval training (22). Overall, there is a complicated and unclear relationship between exercise duration (acute and chronic) and intensity as it relates to inflammatory markers in healthy adults, persons living with CHF, and persons living with other chronic medical conditions (23).

The majority of evidence in the field of exercise science and medicine relates to apparently healthy individuals (24). To date, the most appropriate form of exercise training for persons living with CHF is not known. Traditional rehabilitation for CHF involves low to moderate intensity SS exercise training designed to remain below a symptom threshold. However, several researchers have challenged the traditional rehabilitation model and advocated for the incorporation of HIIT (25–30). These recommendations have been increasingly incorporated into national and international cardiac rehabilitation guidelines (31–34). Although HIIT has been shown to lead to significant benefits in cardiovascular fitness and function and other markers of health status (including Quality of Life), there is still debate regarding the observed benefits in comparison to those seen after traditional SS. Several randomized trials have demonstrated superior cardiovascular and/or health outcomes after HIIT (29, 30, 35, 36), with a potentially greater stimulus to the working muscles (37, 38) in patients living with CHF in comparison to traditional SS training. However, others have demonstrated similar changes in health outcomes after HIIT and SS training in persons living with CHF (39).

To date, limited research has examined the acute effects of HIIT on pro-inflammatory markers. In particular, to our knowledge no study has examined the temporal changes in pro-inflammatory markers in persons living with CHF after HIIT in comparison to traditional SS training. This has important implications for rehabilitation practices for persons living with CHF, as it is critical to ensure an optimal training stimulus while minimizing the risk for over-activation of the inflammatory system. Accordingly, the primary purpose of this study was to examine the effects of HIIT in comparison to traditional SS training on inflammatory biomarkers. We also sought to examine the time course of changes in inflammatory markers following acute bouts of HIIT and SS exercise training. We hypothesized [based on work from apparently healthy individuals (40) and persons living with coronary arterty disease (26)] that HIIT and SS would have similar acute effects on inflammatory markers in CHF and those inflammatory markers would return to baseline levels within 48 h.

## Methodology

### Study Design

**Figure 1:**
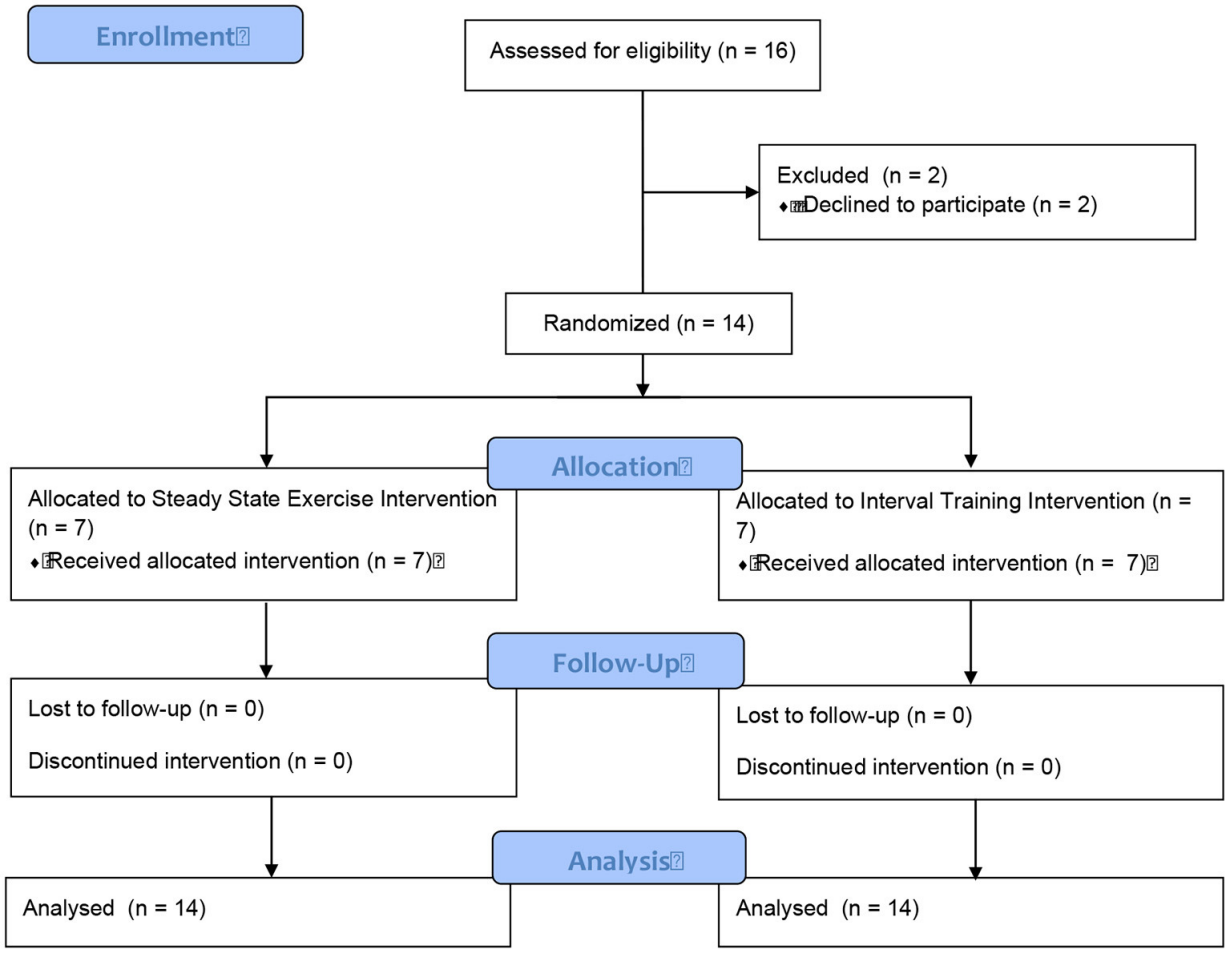
Study flow diagram.

A convenience sample of males living with CHF were recruited from St. Paul's Hospital (Vancouver, Canada) to participate in a randomized controlled study. Sixteen patients were identified as eligible participants and approached regarding the study. Two participants were excluded due to a lack of time to complete the study. Fourteen male participants completed the study assessments (Figure 1). All participants were physically inactive, were over the age of 45 yr, had a peak aerobic power (VO_2_peak) <25 mL·kg^−1^·min^−1^ (7.1 METs), and a left ventricular ejection fraction <35%. Exclusion criteria included: musculoskeletal limitation affecting the ability to use a cycle ergometer, pulmonary disorders that markedly limit exercise, existing contraindications to exercise training, and/or patients who were recently (within last 6 months) involved in an exercise program.

After completing a baseline assessment, participants were stratified (for body mass and VO_2_peak) and randomized to complete a single bout of either a SS or HIIT on a cycle ergometer (Model 818, Stockholm, Sweden) while monitored on a 3-lead telemetry system. The randomization sequence was created with a 1:1 allocation using random block sizes of two by an independent research physician. The treatment allocation was kept blind (via opaque and sealed envelopes) to the research physician and research team until after the randomization procedures.

Participant characteristics are shown in Table 1 and demonstrate that the treatment groups were similar at baseline. All participants gave their written informed consent for inclusion before they participated in the study. The study was conducted in accordance with the Declaration of Helsinki and the protocol was approved by the University of British Columbia (UBC) Providence Health Care Research Ethics Board and the UBC Clinical Research Ethics Board (H05-50260).

**Table 1:** Participant characteristics (values are means ± SD).

**Variable**	**Steady-state training group (*n* = 7)**	**Interval training group (*n* = 7)**
Age (y)	60.1 ± 6.7	57.9 ± 9.8
Height (cm)	177.7 ± 5.3	177.7 ± 5.4
Weight (kg)	96.3 ± 23.4	96.0 ± 13.5
BMI (kg^.^m^2^)	30.2 ± 5.34	30.5 ± 4.6
Ejection Fraction	0.24 ± 0.07	0.30 ± 0.11
VO_2peak_ (mL^.^kg^−1.^min^−1^)	14.9 ± 5.3	12.5 ± 3.9
**Diabetes mellitus**		
Type 1 DM	1	2
Type 2 DM	1	1
**New York heart association class**		
Class 1	2	0
Class 2	4	5
Class 3a	1	2
**Diagnosis**		
Ischemic	3	4
Valvular	1	0
Dilated	1	0
Idiopathic	0	3
**Rhythm**		
NSR	3	6
Atrial fibrillation/flutter	1	0
Paced	3	1
Ventricular pacemaker	2	2
AICD	3	4
**CHF onset (yr)**		
<1 yr	0	1
1–5 yr	5	3
6–10 yr	1	2
11–20 yr	1	1
**NT ProBNP (pg** ^**·**^ **mL** ^**−1**^ **)**		
500–1,000	4	2
1,001–2,000	1	1
2,001–3,000	1	1
3,001–4,000	1	1
4,001–5,000	0	1
5,001–10,000	0	1
>10,000	1	0
**Hemoglobin**		
Low	1	2
Normal	6	5
**Medications**		
ASA	4	4
Beta blocker (CoReg, atenolol, monocor, bisoprolol)	7	6
ARB (atacand)	5	4
Digoxin	4	4
Diuretic (lasix)	6	7
Amiodarone (antiarrhythmic)	1	2
Cholesterol-lowering (lipitor, crestor, simvastatin)	2	3
Anti-coagulant (coumadin)	5	4
Plavix	0	1
ACE inhibitor (altace, captopril, accupril)	3	2
Calcium channel blocker (norvasc)	2	1
Vasodilators (nitroglycerine: patch, spray, tablets; hydralazine)	2	1
Hyperglycemic agents (Metformin, Glyburide, Insulin)	2	3
Other (testosterone, eltroxin, synthroid, pantolec, allopurinol, effexor, welbutrin, celexa, cholchicine, valium, halcion)	5	3

*No significant differences between participants at baseline (p < 0.05)*.

*ARB, angiotensin II receptor blocker; ACE, angiotensin-converting-enzyme inhibitor*.

Participants underwent blood sampling at baseline, and four separate occasions following the training session (immediately post, 6 h post, 24 h post, and 48 h post). Participants were instructed to take all their medications as prescribed and to complete a food record for 3 days prior to testing as well as to complete a food record for the 3 days prior to the training session.

### Sample Size

Our sample size was based on previous investigations from our team examining the effects of HIIT vs. traditional SS aerobic exercise training in persons living with coronary artery disease (*n* = 14) (26). Based on this study, we anticipated that there would be moderate to large effects sizes for transient changes in markers of muscle injury post-training with similar responses between training conditions.

### Exercise Stress Testing

All participants completed an incremental exercise test using an electronically braked cycle ergometer with direct gas monitoring via a calibrated metabolic cart (Vmax, SensorMedics) to assess VO_2_peak (27) (Table 2). The patients completed a symptom-limited incremental exercise protocol (5–10 W/min) with continuous 12-lead electrocardiography, and the assessment of blood pressure and oxygen saturation every 2 min. Criteria for terminating the exercise test included electrocardiogram changes associated with myocardial ischemia, volitional fatigue, a respiratory exchange ratio of >1.1, a leveling off in oxygen consumption, systolic blood pressure > 200 mm Hg, diastolic blood pressure >100 mmHg, dyspnea, or calf/thigh pain (27). The baseline and exercise cardiorespiratory measures are presented in Table 2.

**Table 2:** Cardiorespiratory responses at rest and during incremental to maximal exercise testing (values are means ± SD).

**Variable**	**Steady-state training group (*n* = 7)**	**Interval training group (*n* = 7)**
**Rest**		
Heart rate (bpm)	88.6 ± 21.8	72.7 ± 23.5
Oxyhemoglobin saturation (%)	97.6 ± 1.4	98.3 ± 1.0
Systolic blood pressure (mmHg)	101.3 ± 16.4	105.0 ± 10.8
Diastolic blood pressure (mmHg)	65.7 ± 9.6	68.3 ± 10.5
Oxygen pulse (mL·beat^−1^)	2.7 ± 0.7	3.6 ± 0.9
VO_2_ (mL·kg^−1.^min^−1^)	3.1 ± 0.5	3.6 ± 0.9
VO_2_ (L·min^−1^)	0.3 ± 0.1	0.3 ± 0.1
**Peak exercise**		
Heart rate (bpm)	117.9 ± 17.4	104.4 ± 31.5
Oxyhemoglobin saturation (%)	96.7 ± 2.6	96.0 ± 2.9
Systolic blood pressure (mmHg)	124.9 ± 29.1	136.6 ± 26.6
Diastolic blood pressure (mmHg)	65.7 ± 9.6	68.3 ± 10.5
Oxygen pulse (mL·beat^−1^)	11.6 ± 4.0	11.2 ± 3.8
VO_2peak_ (mL·kg^−1.^min^−1^)	14.9 ± 5.3	12.5 ± 3.9
VO_2peak_ (L·min^−1^)	1.4 ± 0.5	1.1 ± 0.3

*No significant differences between groups*.

### Exercise Protocol

Both exercise training groups engaged in a single supervised exercise training bout on a cycle ergometer (Model 818, Stockholm, Sweden) (Table 3). Participants were monitored with 3-lead telemetry, as well as portable heart rate monitors (PolarTM). Rating of Perceived Exertion (RPE) was collected throughout each exercise bout. Blood pressure was taken before, during, and after the exercise session using an aneroid sphygmomanometer and stethoscope. An equivalent workload was determined for both SS and HIIT in order that total volume of exercise (i.e., isovolumetric) was similar for each group (26, 27) (Table 2). All individuals underwent a standardized 5 min warm-up and a 5 min cool-down prior to, and following, the conditioning exercise. The duration of the conditioning exercise (i.e., 20 min) for both SS and HIIT programs was based on the standard “first” exercise training session in the cardiac rehabilitation program at St. Paul's Hospital.

**Table 3:** Exercise responses during steady state and interval training sessions (values are means ± SD).

**Variable**	**Steady-state training group (*n* = 7)**	**Interval training group (*n* = 7)**
Heart rate (bpm)	105 ± 8	40%: 91 ± 16
		90%: 109 ± 26^*^
		Average: 100 ± 21
Rating of perceived	4.2 ± 0.6	40%: 3.0 ± 1.2
exertion (0–10)		90%: 5.6 ± 1.4^*^
		Average: 4.3 ± 1.2
Work Rate (W)	54.6 ± 25.3	40%: 31.3 ± 11.8
		90%: 70.3 ± 26.5^*^
		Average: 50.8 ± 19.1
METs	2.8 ± 1.0	40%: 1.4 ± 0.4
		90%: 3.2 ± 1.0^*^
		Average: 2.3 ± 0.7
Rate pressure product	13,802 ± 2,664	40%: 10,869 ± 2,317
(mmHg·bpm)		90%: 14,539 ± 4,713^*^
		Average: 12,704 ± 3,490
Total work (J)	65,486 ± 30,388	60,943 ± 22,978

*Average refers to the mean response across the 20 min interval session involving 10 min of high and low intensity exercise (90 and 40%, respectively). Total work was calculated across the 20 min exercise session for both training groups*.

* *Significant (p < 0.05) difference between interval training intensities (i.e., 90 > 40%). No significant difference between training groups*.

### Steady-State Group

The SS group was trained as per the traditional training model (i.e., 65% heart rate reserve/VO_2_ reserve) based on the results of a recent cardiopulmonary exercise test (see Table 3). This is consistent with previous work from our group in individuals with coronary artery disease (20) and CHF (22). The average training METs was 2.8 ± 1.0.

### Interval Training Group

We used the HIIT training protocol (i.e., 2 min work phases at 90% of Heart Rate/VO_2_ reserve and 2 min active recovery bouts at 40% Heart Rate/VO_2_ reserve) that we have previously used safely and effectively in persons living with coronary artery disease (26) and persons living with CHF (27). The average training intensity for each participant in the HIIT group was equivalent to that which would have occurred if the individual had been randomized to the SS group (Table 3). Total work output was calculated as a combination of both the high intensity (i.e., 90% VO_2_peak) and low intensity (i.e., 40% VO_2_peak) phases of the workout (20) (Table 3). The average training METs were 1.4 ± 0.4 and 3.2 ± 1.0, respectively, during the low and high intensity work phases.

### Measurement of Inflammatory Activation

As outlined above, all participants underwent analysis of plasma markers of Tumor Necrosis Factor alpha (TNF-α), Interleukin-6 (IL-6), high sensitivity C-Reactive Protein (CRP), IL-8, and IL-10 at five separate time periods. N-terminal prohormone of brain natriuretic peptide (NT ProBNP) was also assessed. All blood samples were drawn into blood collection tubes from the antecubital vein (with the participant in a seated position) and then immediately immersed in a refrigerated centrifuge and centrifuged within 15 min of collection. Plasma levels of each cytokine (TNF-α, IL-6, IL-8, IL-10, and CRP) and NT ProBNP were measured using a commercially available high-sensitivity ELISA (enzyme-linked immunosorbent assay**)** kits [Biosource (IL-8, IL-10, TNF-α), Biocheck (hsCRP), R & D Systems (IL-6)] according to the manufacturer's instructions.

### Statistical Analyses

The baseline characteristics and physiological responses to exercise were evaluated using paired *t*-tests. Changes in plasma cytokine markers at baseline and at four time periods (i.e., immediately, 6, 12, and 24 h) following a single bout of either ST or HIIT exercise were examined using a mixed model Analysis of Variance with Tukey *post-hoc* comparisons. The relationship between various physiological parameters of interest and baseline levels of inflammatory and skeletal muscle injury markers were determined by Pearson Correlation Coefficient. The level of significance was set *a priori* at *p* < 0.05. All data are presented as means ± SD.

## Results

There were no significant differences in baseline physiological characteristics between the randomized SS and HIIT groups (Table 1). Similarly, there were no significant differences between groups with respect to the cardiorespiratory responses at rest and during incremental to maximal exercise testing (Table 2).

By design, there was a significant difference between the 40 and 90% interval stages for heart rate, work rate, METs, Rate Pressure Product, and Total Work. When comparing the average values across the entire SS and HIIT sessions there was no significant differences in heart rate, work rate, METs, Rate Pressure Product, and Total Work (Table 3).

**Figure 2:**
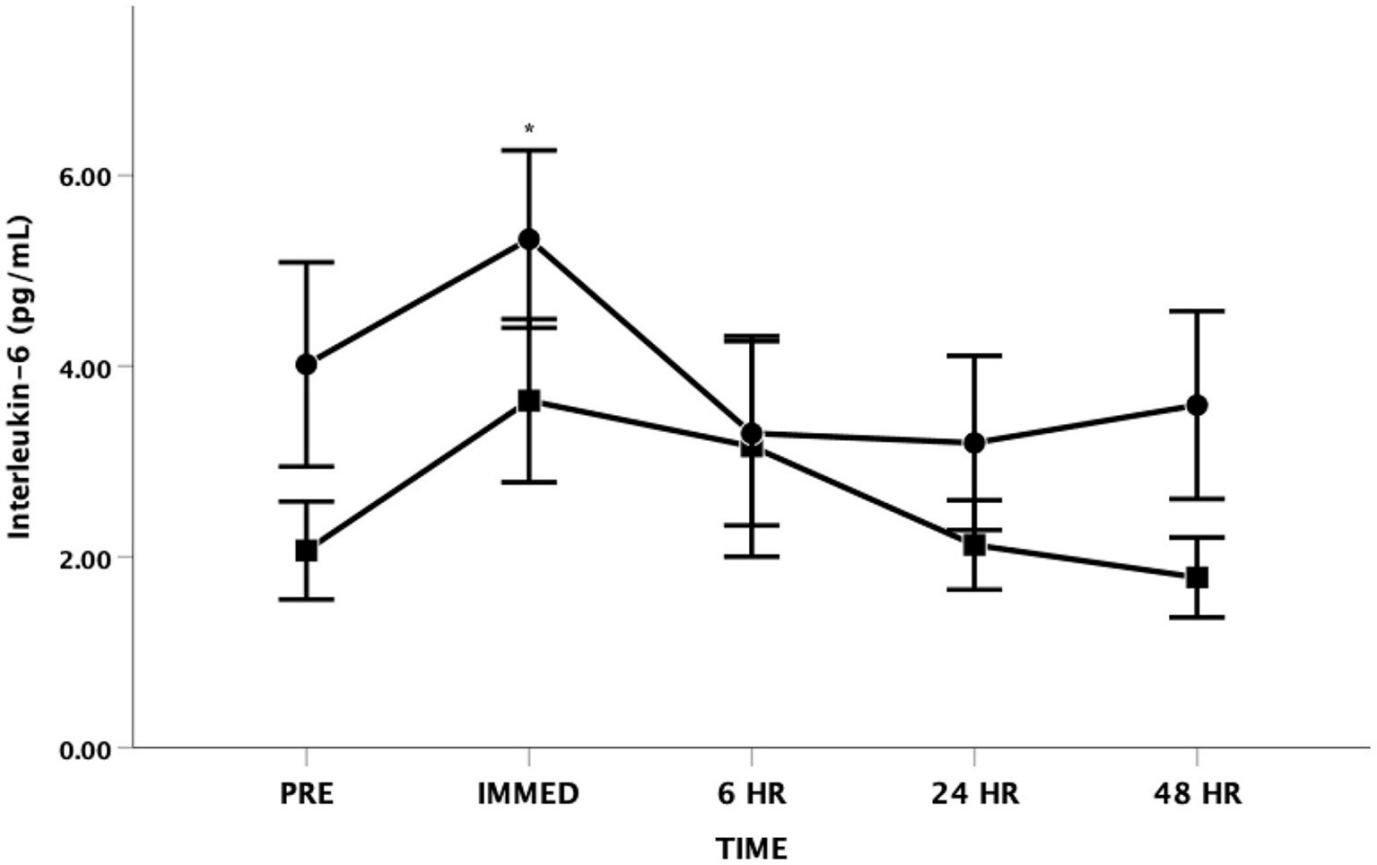
Interleukin-6 (IL-6) as a function of time. Means ± SD. *Main effect for time observed immediately post-exercise (*p* < 0.05). Circle = Steady State; Square = Interval. Error Bars = ± SEM.

There was a main effect for changes in IL-6 with time [*F*_(4, 48)_ = 3.484, *p* = 0.014, Partial Eta = 0.225; Observed Power = 0.825] (Figure 2). The IL-6 increased from baseline to immediately post and then returned to near baseline levels at all other time points. There was no statistical significance in IL-6 levels between groups [*F*_(1, 12)_ = 1.803, *p* = 0.204, Partial Eta = 0.131; Observed Power = 0.235] and there was no significant time by group interaction effect [*F*_(4, 48)_ = 0.874, *p* = 0.486, Partial Eta = 0.068; Observed Power = 0.257].

**Figure 3:**
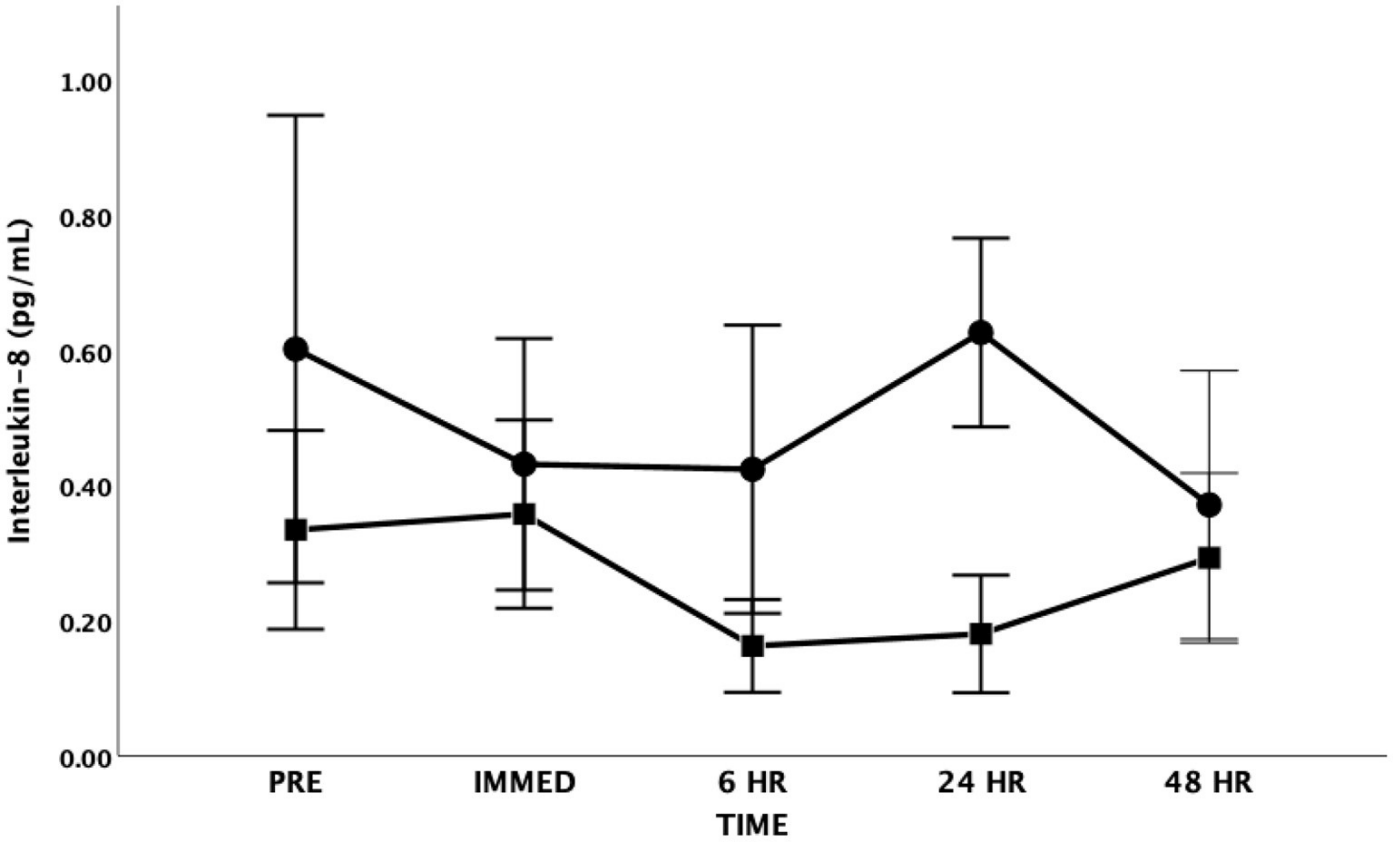
Interleukin-8 (IL-8) as a function of time. Means ± SD. Circle = Steady State; Square = Interval. Error Bars = ± SEM.

There were no significant changes in IL-8 following the exercise bout in either the SS or HIIT groups [*F*_(4, 48)_ = 1.339, *p* = 0.269, Partial Eta = 0.100; Observed Power = 0.386] (Figure 3). There was also no significant differences between groups [*F*_(1, 12)_ = 0.931, *p* = 0.354, Partial Eta = 0.072; Observed Power = 0.144] and there was no significant time by group interaction effect [*F*_(4, 48)_ = 1.759, *p* = 0.153, Partial Eta = 0.128; Observed Power = 0.497].

**Figure 4:**
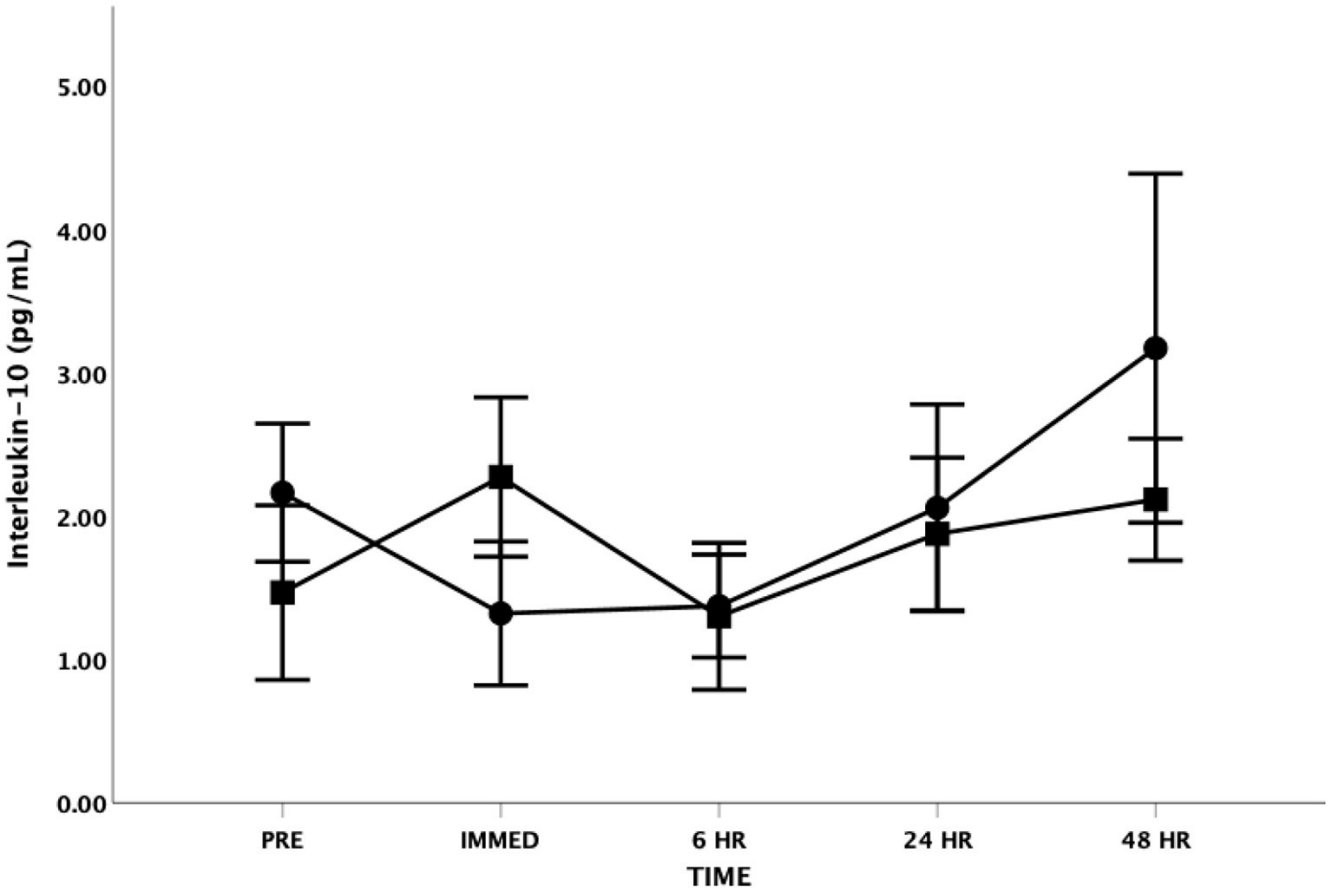
Interleukin-10 (IL-10) as a function of time. Means ± SD. Circle = Steady State; Square = Interval. Error Bars = ± SEM.

For IL-10, the Mauchly's Test of Sphericity was statistically significant indicating that the variances of the differences were not equal for the main effect of time. Accordingly, the Greenhouse-Geisser correction was applied revealing no significant main effect for time [*F*_(1.7, 19.8)_ = 2.861, *p* = 0.089, Partial Eta = 0.193; Observed Power = 0.455] (Figure 4). There was also no significant difference between groups [*F*_(1, 12)_ = 0.080, *p* = 0.782, Partial Eta = 0.007; Observed Power = 0.058] and no significant time by group interaction effect [*F*_(1.7, 19.8)_ = 1.863, *p* = 0.185, Partial Eta = 0.134; Observed Power = 0.314].

**Figure 5:**
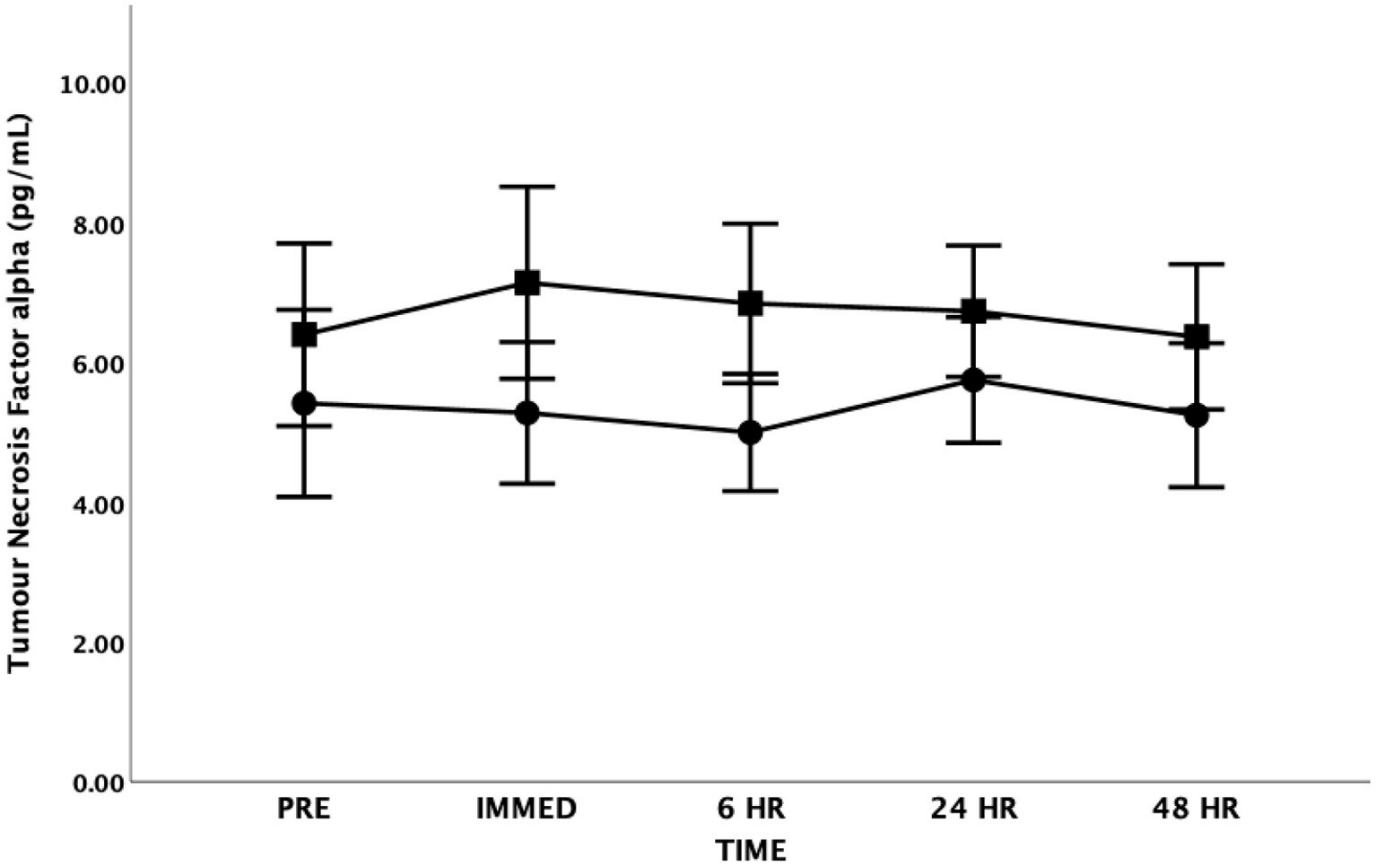
Tumor Necrosis Factor-alpha (TNF-α) as a function of time. Means ± SD. Circle = Steady State; Square = Interval. Error Bars = ± SEM.

There were no significant differences in TNF-α at any time point [*F*_(4, 48)_ = 0.305, *p* = 0.873, Partial Eta = 0.025; Observed Power = 0.112] or between groups [*F*_(1, 12)_ = 0.902, p = 0.361, Partial Eta = 0.070; Observed Power = 0.141] (Figure 5). There was also no interaction effect between time and group [*F*_(4, 48)_ = 0.412, *p* = 0.799, Partial Eta = 0.033; Observed Power = 0.137].

**Figure 6:**
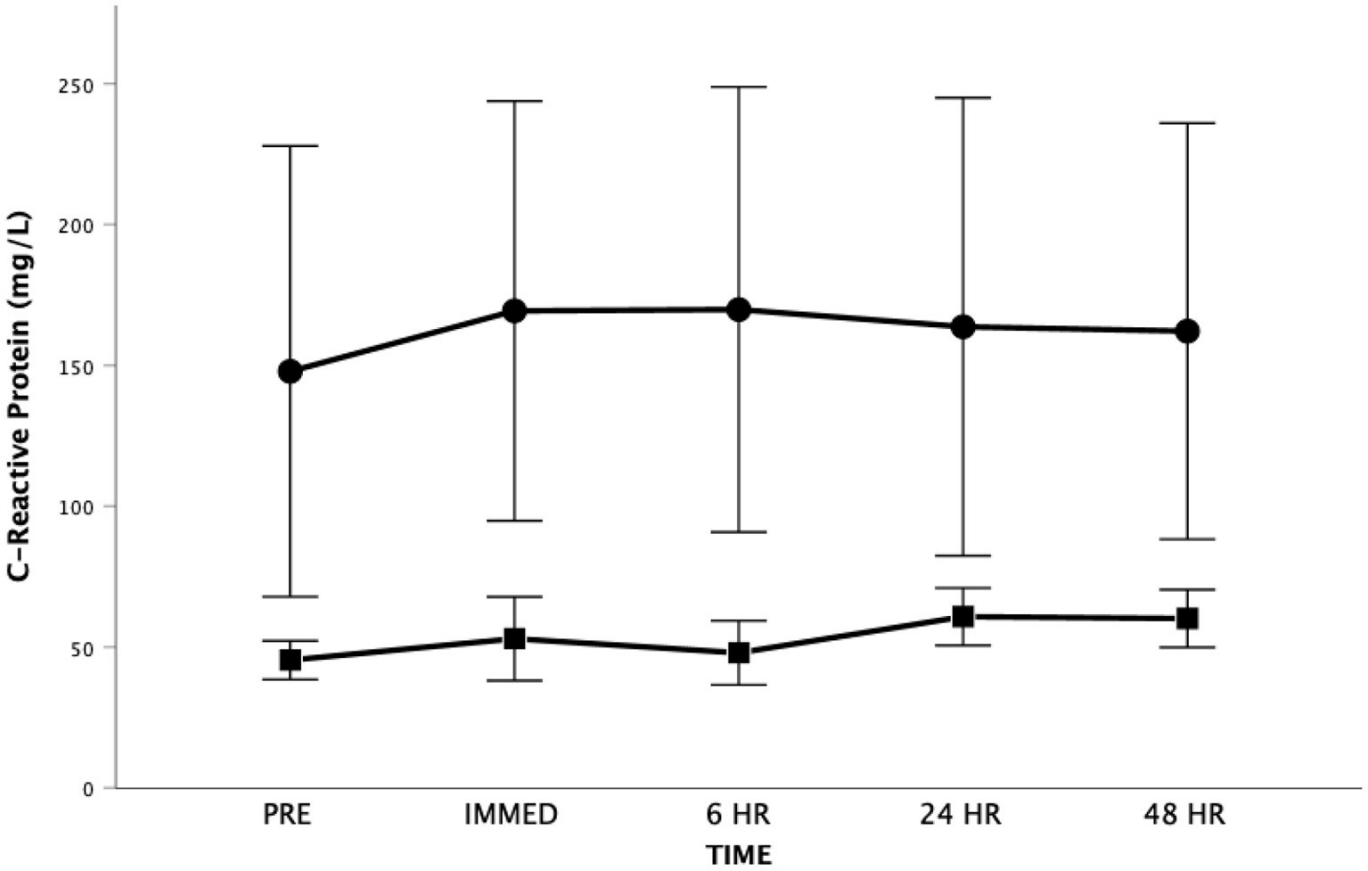
High sensitivity C-Reactive Protein as a function of time. Means ± SD. Circle = Steady State; Square = Interval. Error Bars = ± SEM.

For CRP, the Mauchly's Test of Sphericity was statistically significant indicating that the variances of the differences were not equal for the main effect of time. Accordingly, the Greenhouse-Geisser correction was applied revealing no significant main effect for time [*F*_(1.5, 18.4)_ = 0.855, *p* = 0.414, Partial Eta = 0.066; Observed Power = 0.160] (Figure 6). There was also no significant difference between groups [*F*_(1, 12)_ = 1.980, *p* = 0.185, Partial Eta = 0.142; Observed Power = 0.254] and no significant time by group interaction effect [*F*_(1.5, 18.4)_ = 0.442, *p* = 0.598, Partial Eta = 0.036; Observed Power = 0.105]. Both groups had baseline CRP levels that would be considered above normal values. It should be highlighted that one participant had high sensitivity CRP levels that approximated 600 mg/L at all time periods (explaining the variance seen in this measure).

Pearson Correlation Coefficients were determined for physiological variables of interest and markers of inflammation at baseline (see Tables 4A, 4B). There were significant correlations (*p* < 0.05) for IL-6 and CRP (0.65), IL-8 and CRP (0.81), and IL-6 and IL-8 (0.63). There was also a significant correlation between participant height and weight (0.57).

**Table 4A:** Baseline relationship between clinical indicators.

	**Ejection Fraction**	**Oxygen Consumption**	**Height**	**Weight**	**NT Pro BNP**	**Age**
Ejection Fraction	1.00	0.01	−0.05	−0.11	0.18	−0.08
Oxygen Consumption	0.01	1.00	0.23	0.23	0.04	0.37
Height	−0.05	0.23	1.00	0.57^*^	0.45	0.27
Weight	−0.11	0.23	0.57	1.00	−0.29	−0.30
NT ProBNP	0.18	0.04	0.45	−0.29	1.00	0.44
Age	−0.08	0.37	0.27	−0.30	0.44	1.00

* *Marked correlations are significant at p < 0.05. The heat map correlation is designed such that a correlation of −1 is represented with red, a correlation of 0 (mid-point) is represented with yellow, and a correlation of +1 is represented with a color of dark green*.

**Table 4B:** The baseline relationship between various inflammatory biomarkers.

	**CRP**	**IL-6**	**IL-8**	**IL-10**	**TNF-α**
CRP	1.00	0.65^*^	0.81^*^	0.43	0.44
IL-6	0.65^*^	1.00	0.63^*^	0.27	0.41
IL-8	0.81^*^	0.63^*^	1.00	0.49	0.41
IL-10	0.43	0.27	0.49	1.00	0.36
TNF-α	0.44	0.41	0.41	0.36	1.00

*NT ProBNP, N-terminal pro b-type natriuretic; CRP, high sensitivity C-Reactive protein; IL-6, Interleukin 6; IL-8, Interleukin 8; IL-10 Interleukin 10; TNF-α, Tumor Necrosis Factor alpha*.

* *Marked correlations are significant at p < 0.05. The heat map correlation is designed such that a correlation of −1 is represented with red, a correlation of 0 (mid-point) is represented with yellow, and a correlation of +1 is represented with a color of dark green*.

## Discussion

To our knowledge this study is the first randomized trial to evaluate the acute and temporal effects of HIIT in comparison to traditional SS exercise training on markers of inflammatory function in persons living with CHF. The present study confirmed our hypothesis and demonstrated that there were no differences between the HIIT group and the SS training group for markers of inflammatory activation following either acute SS or HIIT training in inactive and low fitness persons living with CHF. Our secondary hypothesis was also supported with the inflammatory markers returning to baseline within 48 h.

Chronic systemic inflammation is commonly observed with physical inactivity, obesity, and in persons living with chronic medical conditions (such as cancer and diseases of the cardiovascular system) (41). Chronic inflammation has been linked to the development of insulin resistance, tumor growth, and atherosclerosis (41). Accordingly, a growing body of research has examined the role of regular physical activity and exercise training on markers of systemic inflammation in apparently healthy individuals and those living with chronic medical conditions (41, 42).

Examining the acute effects of exercise provides unique insight into the potential health benefits of exercise in persons living with CHF. It is important to highlight that compelling research has revealed that skeletal muscle can produce and secrete cytokines [termed “myokines” (43)] that apply autocrine, paracrine, and/or endocrine effects (41, 43). Contractile activity appears to be the key regulatory factor for the expression and secretion of myokines (41).

Interleukin-6 is produced by several cells such as stimulated monocytes/macrophages, fibroblasts, endothelial cells, and skeletal muscle fibers (41). Interleukin-6 was traditionally classified as a pro-inflammatory cytokine with its elevation being commonly associated with systemic inflammation and insulin resistance; however, more recently its anti-inflammatory properties (particularly during exercise conditions) have also been highlighted (41).

Our baseline IL-6 values were elevated in both HIIT and SS groups in comparison to what is often observed in apparently healthy adults supporting data from other studies in persons living with CHF (44–49). In persons living with CHF, elevated IL-6 has been related to impaired cardiac function (e.g., lowered ejection fraction), decreased cardiac functional class, muscle wasting, poor exercise tolerance, the degree of neurohumoral activation, and/or the progression and deterioration of CHF (44, 50–52).

It is important to acknowledge the potential anti-inflammatory effects of IL-6 release during exercise conditions. Interleukin-6 can be classified as a myokine as it is produced by contracting skeletal muscles and is released into the circulation in large quantities (41, 53). Plasma IL-6 is generally thought to increase in an exponential fashion with exercise (54). It is not uncommon for IL-6 levels to increase more than 100-fold with strenuous exercise then rapidly return to baseline conditions (55). As reviewed by Febbraio and Pedersen (55) the appearance of IL-6 in blood precedes other cytokines and exhibits the greatest changes. The IL-6 temporal response is related to exercise intensity, duration, the muscle mass recruited, and endurance capacity (53, 54, 56). The temporal response (kinetics) appears to differ between the type of muscle concentration (i.e., concentric vs. eccentric) (55). Originally the exercise-related increase in IL-6 was thought to be the result of muscle damage. However, research has demonstrated marked post-exercise increases in IL-6 independent of markers of muscle damage (41, 57, 58).

Our findings are consistent with previous research that demonstrates IL-6 increases in response to concentric muscle actions (such as employed during cycle ergometry), However, we did not observe as great of increases in exercise IL-6 as seen by other researchers examining the IL-6 response to maximal exercise in persons living with CHF (48, 49). These differences may be related to the relatively short duration of exercise in our study and the average training intensity employed. Our findings are supported by a recent study (59) that compared light intensity vs. moderate intensity SS exercise in persons living with CHF; overall, the light intensity had lower IL-6 following the session. However, 1 h post-exercise there was an increase in IL-6 similar to the current study. In our study, the average training intensity was controlled between the HIIT and SS groups, further highlighting the importance of understanding exercise intensity as it relates to inflammatory markers in persons living with CHF.

Pedersen et al. highlight that IL-6 plays several important biological roles including the induction of lipolysis, the suppression of TNF-α production, and the stimulation of cortisol production (53). Schnyder and Handschin (41) argued that the exercise-related release of IL-6 has pleiotropic effects by increasing glucose uptake and fatty acid oxidation and enhanced insulin secretion that supports increased glucose uptake into the working muscles.

Several authors have emphasized the important anti-inflammatory roles IL-6 may play during exercise conditions (41, 53, 55). Investigators have suggested that elevations in IL-6 in response to exercise may play an important anti-inflammatory role by inhibiting the production of TNF-α (54). Importantly, TNF-α has direct inhibitory effects on insulin signaling and the ability of IL-6 to inhibit TNF-α production may represent an important mechanism whereby exercise enhances insulin sensitivity (56). In the current study, although IL-6 increased immediately post-exercise, TNF-α did not significantly change following either SS or HIIT. It is possible that IL-6 had an inhibitory effect on TNF-α in the current study as suggested by other researchers (56, 60). This is consistent with other researchers who report that maximal IL-6 levels are found immediately after the exercise followed by a rapid decline (54, 55). Our findings are supported by a review paper on cytokine kinetics that reported more than half of the studies examining TNF-α could not confirm significant increases after exercise (61). However, other researchers have found an increase in both IL-6 and TNF-α levels immediately following maximal exercise in both patients with mild to moderate CHF and in normal controls (49). These researchers concluded that increases in basal IL-6 and TNF-α levels are associated with high sympathetic nervous system activity and exercise intolerance in patients with mild to moderate CHF.

It should be acknowledged that other factors may play a role in the lack of change in TNF-α levels in either the SS or the HIIT group at any time point following the exercise bout. Suzuki et al. highlight that TNF-α is rapidly cleared from the circulation into the urine as a result of a short half-life (14–18 min) (61). If TNF-α is rapidly expelled into the urine it is possible that the immediate post-blood sample taken in our study within 1 h of cessation of exercise would have missed this window especially if the increase in TNF-α levels were relatively small given the duration and intensity of each exercise bout. Furthermore, as noted by Lee et al. (62) the lack of changes in TNF-α seen following exercise may be due to the measurement method; skeletal muscle biopsy may show a different story, which would be important to understanding the effects of inflammatory markers on exercise capacity for adults living with CHF.

In the current study, the baseline TNF-α levels were elevated when compared to data from apparently healthy controls (63) research (5.9 ± 3.3 vs. 2.5 ± 1.8 pg·mL^−1^). These findings are consistent with other research that has reported increased TNF-α levels in persons living with CHF (45, 46, 49, 63). Our findings support the work of others highlighting the systemic inflammation that presents in persons living with CHF.

Interleukin-8 is a chemokine secreted by several cell types (such as monocytes, neutrophils, epithelial cells, fibroblasts, endothelial cells, mesothelial cells, and tumor cells) (64). It is a chemoattractant and activator of neutrophils (and other immune cells) that is released in response to several stimuli (such as sheer stress, ischemia, hypoxia, and stimuli that activate the nuclear factor (NF)-κB pathway) (64, 65). Interleukin-8 is also associated with the promotion of angiogenesis (41).

Elevated IL-8 levels are commonly observed in chronic medical conditions associated with systemic inflammation. Also, elevated IL-8 has been associated with poor medical outcomes in persons living with CHF (65). Previous studies have reported elevated IL-8 levels in persons living with CHF in comparison to apparently healthy controls (66, 67). For instance, Larsen et al. reported that IL-8 levels were markedly elevated in males with New York Heart Association class II to III stable, ischemic CHF (mean age 67 ± 8 y) when compared to age and sex-matched healthy controls (67). The CHF group had IL-8 levels of 12.7 ± 9.2 pg·mL^−1^, whereas there was no detectable level of IL-8 in the healthy controls. In our current study, the IL-8 levels were detectable; however, they were not elevated to the same extent especially in the presence of elevated baseline levels of other cytokines (i.e., TNF-α, IL-6, and CRP). It is possible that etiology of CHF may have an influence on the expression of this cytokine.

Elevated IL-8 levels have been observed after exercise, particularly exercise that involves both eccentric and concentric phases (such as running) (68). Marked increases in IL-8 (e.g., 7–11-fold) immediately following prolonged strenuous events (such as marathons and half-marathons) have also been observed in apparently healthy adults (69, 70). Interestingly, other researchers have found no (or limited) change in IL-8 following largely concentric exercise (e.g., rowing or cycle ergometry) (71, 72). In fact, eccentric exercise appears to have a greater effect on IL-8 than concentric exercise (41).

In our current study, there were no significant changes in IL-8 after both SS and INT in persons living with CHF. This is consistent with other researchers that have examined IL-8 after concentric exercise in apparently healthy individuals (71, 72). This is likely the result of the relatively short duration (i.e., 20 min) of exercise, the overall lower intensity of exercise, and the nature of the muscle contractions (i.e., concentric) during bicycle ergometry (41).

Interleukin-10 is believed to be one of the most important and potent anti-inflammatory cytokines in CHF (44). It is known to down-regulate the production of TNF-α and IL-6 and other cell-mediated immune responses (44). Circulating IL-10 concentrations have been reported to be either increased or decreased in CHF patients compared to apparently healthy age-matched controls (63, 73, 74). For instance, Stumpf et al. looked at serum levels of IL-10 in patients with advanced CHF compared to age-matched controls and found significantly reduced plasma levels of IL-10 (2.3 ± 1.9 pg·mL^−1^ compared to healthy controls 5.2 ± 2.3) (63). Similarly, in our current study we observed reduced IL-10 levels (1.8 ± 1.4 pg·mL^−1^) when compared to the healthy controls from the research by Stumpf et al. These authors also looked at the ratio of TNF-α to IL-10 and found it to be significantly higher in the CHF group vs. the control group (3.2 ± 1.2 vs. 0.4 ± 0.2). In our current research, the ratio of TNF-α to IL-10 was very similar to that of Stumpf et al. (3.2 ± 2.4) indicating an immunological imbalance in favor of inflammation in our participants living with CHF (63).

Activity-induced IL-10 is produced by stretching and compressing the epithelial cells (75), as well as in response to high levels of other pro-inflammatory markers (such as IL-6 and TNF-α). Other researchers report the relative change in IL-10 following strenuous exercise follows a similar time-course as IL-6, albeit, to a lesser degree (76) and with a slight delay in the response (24). However, there is no clear consensus on the effect of strenuous exercise on IL-10 (24). Some authors report significant increases in IL-10 following strenuous exercise (77–80) while others report limited change (15, 81). A recent systematic review revealed that the changes in IL-10 after intense exercise in healthy adults ranged from 1.57 to 32.99 times (24). Researchers have argued that exercise intensity is a key determinant of the IL-10 response to acute exercise (79) with the greatest changes being seen after strenuous high intensity exercise (24); however, a recent systematic review in healthy individuals revealed that exercise duration and not intensity was the most important predictor of exercise-related changes in IL-10 following acute exercise (40). In our current study, IL-10 did not change significantly following either SS or HIIT exercise. The average relative intensity of both the SS and HIIT exercise bouts would be considered moderate intensity. This in combination with the short duration of the training sessions (both SS and HIIT) in our study supports previous research in healthy individuals (15, 24).

C-Reactive Protein is produced by hepatocytes in response to a variety of inflammatory cytokines (e.g., IL-6) and has been shown to be a non-specific marker of systemic inflammation (82). The serum concentration of high sensitivity CRP is known to be an independent predictor of adverse cardiovascular events, including death, the need for transplantation and worsening CHF requiring hospitalization (83–86). A recent study (87) involving persons living with CHF revealed that CRP was associated with reduced exercise tolerance (*r* = −0.65), lower VO_2_ at the anaerobic threshold (*r* = −0.66), and lower VO_2_peak (*R* = −0.70), reflecting worsened cardiovascular performance. In the current study, the baseline high sensitivity CRP values were elevated (Figure 6) possibly reflecting the severity of the heart dysfunction and/or other comorbid conditions that accompany CHF in our participants.

Research on the effects of strenuous exercise on CRP levels is limited particularly in persons living with CHF. As reviewed by Cerqueira et al. (24) strenuous exercise is generally associated with an increase in CRP in healthy individuals that peaks 24 h or more after exercise with a greater increase after high intensity exercise. However, the results in healthy and in particular CHF participants are somewhat equivocal. In our study, CRP did not show any statistically significant change over all time periods or between the SS or HIIT groups. Similar equivocal findings are seen when comparing the changes in CRP that are seen with exercise training. For instance, a recent meta-analysis failed to find evidence of effects of exercise interventions on CRP in persons living with CHF (19). This is contrary to another meta-analysis (88) that revealed a small and significant decrease in CRP after exercise training in healthy adults.

Significant correlations between CRP and IL-6 and IL-8 and IL-6 and IL-8, respectively, were found in the current study. These relationships are not surprising given that CRP and IL-6 have both been associated with increased severity of left ventricular dysfunction and increased NYHA functional class and that high levels of IL-8 have been shown to predict CHF in patients following anterior myocardial infarction (51, 59, 89, 90). All participants in the current study had ejection fractions <35% and were primarily classified in the NYHA functional classes II to III, indicating moderate to severe CHF. Additional support for this finding is data from studies suggesting that CRP may function to markedly exaggerate the actions of IL-6 from endothelial cells and therefore, the increased vascular production of IL-6 may represent a positive feedback loop for the continued production of CRP from the liver (76, 91, 92).

### Limitations

It is important to acknowledge the limitations of this randomized clinical trial. For instance, the small sample size limits the generalizability to like-patients (i.e., persons living with CHF with New York Heart Association Classification of Class I-III that have low aerobic fitness levels). Furthermore, because of the convenience sample, it is likely that there is some impact of selection bias and participant selection, consistent with human experimentation in clinical settings. It could be argued that since the participants self-selected to participate in an exercise intervention that they may represent a healthier or more health-conscious group of persons living with CHF. As such, the levels of inflammatory markers may not be representative of all persons living with CHF. However, it should be highlighted that the aerobic fitness levels (VO_2_peak = 13.6 ± 4.6 mL·kg^−1^·min^−1^; METs = 3.9 ± 1.3) and clinical characteristics of the participants are consistent of persons living with CHF (27) that have a higher risk for premature mortality.

Consistent with the field of research, we opted to conduct unadjusted statistical analyses in this investigation and therefore, we did not control for confounding factors, such as variation in age, medications and their interactions, variation in CHF class, and other disease status. Due to the small sample size, it is unlikely that any confounders would have demonstrated important adjustments to the data. Moreover, stratified sampling procedures were used to help reduce the variability between groups. This study examined the effects of an acute bout of exercise, which does not generalize to long-term effects of exercise on inflammatory markers in adults living with CHF; however, it is important to understand inflammatory profiles at various intensities and duration to understand the profiles of inflammatory markers in this high-risk group that stands to benefit greatly from exercise.

## Conclusions

Exercise training plays an essential role in the optimal treatment of patients living with CHF with clear evidence of an overall reduction in premature mortality. There is evidence suggesting that inflammatory status plays a key role in determining the responsiveness to exercise training in persons living with CHF (14, 22). Currently, it is not clear the effects HIIT on inflammation and the time course of inflammatory marker changes following HIIT and SS exercise. The results of this investigation suggest that when either SS or HIIT exercise is prescribed at a similar volume of exercise on a cycle ergometer, there is no indication of excessive or differential activation of the inflammatory system. This research has important implications for persons living with CHF, practitioners, and cardiac rehabilitation practices. The finding of no significant difference in inflammatory activation between SS and HIIT supports the inclusion of HIIT in the menu of activities provided to persons living with CHF.

Further research is warranted to examine the long-term effects of SS or HIIT exercise training in persons living with CHF. Future research should examine the effects of various modalities, dosages, and intensities of exercise on inflammatory markers to further improve clinical exercise prescriptions for person living with CHF.

## Data Availability Statement

The raw data supporting the conclusions of this article will be made available by the authors, without undue reservation.

## Ethics Statement

The studies involving human participants were reviewed and approved by the University of British Columbia Providence Health Care Research Ethics Board and the University of British Columbia Clinical Research Ethics Board (H05-50260). The patients/participants provided their written informed consent to participate in this study.

## Author Contributions

AT, AI, JH, and DW: conceptualization, methodology, and investigation. AT, ES, SB, and DW: formal analysis and data curation. AI, JH, SB, and DW: resources. AT and DW: writing—original draft preparation, visualization, and project administration. AT, AI, ES, JH, SB, and DW: writing—review and editing. AI and DW: supervision. DW: funding acquisition. All authors contributed to the article and approved the submitted version.

## Funding

This research was supported by the Canada Foundation for Innovation (Project #7611), the BC Knowledge Development Fund (Project #7611), the Michael Smith Foundation for Health Research (Scholar Award to DW), the Canadian Institutes of Health Research (No. IA5-156528), and the Natural Sciences and Engineering Research Council of Canada (No. NSERC RGPIN-2018-04613). AT received graduate support from the University of British Columbia.

## Conflict of Interest

The authors declare that the research was conducted in the absence of any commercial or financial relationships that could be construed as a potential conflict of interest.

## Publisher's Note

All claims expressed in this article are solely those of the authors and do not necessarily represent those of their affiliated organizations, or those of the publisher, the editors and the reviewers. Any product that may be evaluated in this article, or claim that may be made by its manufacturer, is not guaranteed or endorsed by the publisher.
